# Editorial: The link between metabolic syndrome and chronic kidney disease: Focus on diagnosis and therapeutics

**DOI:** 10.3389/fendo.2022.1030497

**Published:** 2022-09-14

**Authors:** Ningning Hou, Congjuan Luo, Guiting Lin

**Affiliations:** ^1^ Department of Endocrinology and Metabolism, Affiliated Hospital of Weifang Medical University, Weifang, China; ^2^ Clinical Research Center, Affiliated Hospital of Weifang Medical University, Weifang, China; ^3^ Department of Nephrology, The Affiliated Hospital of Qingdao University, Qingdao, China; ^4^ Knuppe Molecular Urology Laboratory, Department of Urology, University of California, San Francisco, San Francisco, CA, United States

**Keywords:** metabolic syndrome, obesity, chronic kidney disease, diagnosis, therapeutics

Metabolic syndrome (MS), a summation of interrelated disorders (obesity, dyslipidemia, hyperglycemia and hyperuricemia, etc.), is related to proteinuria and incident chronic kidney disease (CKD). These metabolic disorders lead to various kidney dysfunction, including obesity-related kidney disease (ORKD), diabetic kidney disease (DKD), gouty nephropathy and even end-stage renal disease (ESRD). The increasing prevalence of MS attracts much attention to MS-related renal injury. However, the pathobiology and pathophysiology of kidney injury are different due to various metabolic risk factors; this increases the difficulty of diagnosis and therapy for MS-induced CKD. Thus, studies on interactions of MS-related diseases with CKD are essential for a better understanding of diagnosis and therapeutic strategies for metabolic-induced CKD. This Research Topic provides a platform for recent advances in diagnosis and therapeutic strategies for MS-related CKD. The special issue represents a collection of 6 original research articles and 5 review articles ranging from laboratory to patient-oriented studies.

Due to metabolic disorders and impaired renal function, the incidence of MS is significantly more prevalent in CKD patients than in the general population. Thus, MS has become an obvious risk factor for CKD. Lin et al. provide a comprehensive discussion on the diagnosis and treatment of MS and CKD. This review presents a comparison of several MS criteria and analyzes their differences. The authors summarize the epidemiology, pathogenesis, diagnosis, and treatment advances of MS and MetS-related renal injury. And interventions for MetS-related kidney damage are also the focus of this review.

Obesity, the hallmark characteristic of MS, has become a worldwide epidemic associated with several complications. Adipose accumulation and renal lipotoxicity lead to inflammation and glomerular injury. Original research studies by Ye et al. showed that empagliflozin, sodium-glucose cotransporter 2 inhibitor, reduced obesity-renal injury by activating heme oxygenase-1(HO-1)–adiponectin axis. Transcriptome analysis indicated that empagliflozin influences key genes closely related to inflammation and NLRP3 inflammasome. The study provides new knowledge concerning potential targets for ORCD. Additionally, obesity is related to many adverse pregnancy outcomes and health status of offspring. The review by Wei et al. concluded the role of obesity in infertility development, fetus growth, the health of offspring and the occurrence of CKD. Meanwhile, they also outlined the therapeutic effect of weight loss on pregnancy and ORCD.

DKD, the most devastating microvascular comorbidity of diabetes, has been the leading severe cause of ESRD. Screening and identifying special biomarkers for diagnosis and therapeutic of DKD is essential. Using bioinformatics algorithms (WGCNA, LASSO, SVM-RFE and RF) and Venn diagrams, Han et al. finalized two powerful genes relevant to infiltrating immune cells (PRKAR2B and TGFBI) as diagnostic biomarkers for DKD, which were further validated in the test data. They developed a diagnostic model that combines these two genes to assess the risk of glomerular injury. Another research group, Wei et al., identified 176 up-regulated and 91 down-regulated genes by building a protein interaction network to select hub pathogenic genes. Four of these hub genes, FOS, EGR1, ATF3 and JUN, were closely linked to immune response or inflammatory genes in early DKD. These two studies demonstrate that immune response or inflammatory genes are associated with DKD.

Patients with ESRD are at risk for various complications and therapy difficulties. For example, uremic peripheral neuropathy is a serious neurological complication in CKD stage 5 dialysis (CKD5D). Li et al. studied the use of shear wave elastography (SWE) in diagnosing peripheral neuropathy in hemodialysis patients (CKD5D). The authors carried out Young’s modulus measurements of the tibial nerve. It was found that the Young’s modulus of the tibial nerve was 48.35 kPa, which is the best threshold for diagnosing uremic peripheral neuropathy in CKD5D.

Besides diagnosis, the optimal treatment for DKD also remains a major challenge. TangShenWeiNing formula (TSWN) is an expertly developed traditional Chinese herbal formula. And TSWN has been used clinically for over 20 years to treat DKD. The original article by Chang et al. revealed that TSWN reduced albuminuria and renal fibrosis and prevented renal cell apoptosis by modulating SIRT1/HIF-1a signaling in diabetic mice kidneys. This result indicates that TSWN has a significant protective effect on DKD therapy.

Bone, as a powerful organ, has a powerful endocrine function. Bone cells in the skeleton help regulate phosphorus balance, and the proteins produced by bone cells can influence insulin secretion and regulate glucose metabolism. Focusing on bone-derived hormones such as fibroblast growth factor 23, osteocalcin, sclerostin and lipocalin 2, Li et al. summarize their roles in regulating glucose metabolism and DKD. They concluded that bone-derived hormones are therapeutic targets for diabetes and its complications and are closely tied to insulin secretory, insulin resistance and glucose metabolism.

Peritoneal dialysis is one of the most commonly used alternative therapies for patients with ESRD. However, it has been suggested that urgent initiation of peritoneal dialysis (USPD) may carry the risk of catheter mobilization and dialysate outflow. Hu et al., discussed the safety and feasibility of a ≤24-hour break-in period for diabetic patients receiving USPD. This real-world study found that Break-in Period ≤24 h was not an independent risk factor for complications and technical failure compared to diabetic patients with a Break-in Period >24 h after catheter implantation to start peritoneal dialysis. Therefore, Break-in Period ≤ 24 hours for USPD initiation may be safe and feasible for patients with ESRD.

Primary cilia are a class of organelles that protrude from the surface of eukaryotic cells and have microtubule-based structures that can sense various pericellular signals. In a Mini-Review, Bai et al. focused on the relationship between cilia defects and kidney disease. Studies show that HDAC6, a key regulator of glomerular hyperfiltration-induced cilia breakdown, is downregulated, promoting cilia elongation and accelerating the progression of DKD. Remote control of ciliary motility by lipid nanoparticles targeting renal cilia would be a possible therapeutic target for DKD.

Hyperuricemia, caused by an increase in uric acid, usually leads to gouty nephropathy and increases in severity with the deterioration of kidney function. However, urates might have protection effects *via* their antioxidant properties. Mei et al. focus on the role of uric acid and gout in kidney disease and the problems encountered in the current treatment of gouty kidney disease. Hyperuricemia may participate in CKD development and progression, and uric acid-lowering therapy may slow CKD progression.

In summary, this Research Topic highlights the critical role of various metabolic risks in the progression, diagnosis and treatment of CKD. Metabolic risks have a significant negative impact on CKD. More in-depth research is required to explore the new diagnosis and therapy strategies for MS-related CKD.

## Author contributions

All authors listed have made a substantial, direct, and intellectual contribution to the work and approved it for publication.

## Funding

This study was supported by grants from the National Natural Science Foundation of China (81870593 and 82170865), Natural Science Foundation of Shandong Province of China (ZR2020MH106).

## Acknowledgments

We are grateful to all the authors and reviewers for their excellent contributions and insightful comments to this Research Topic.

## Conflict of interest

The authors declare that the research was conducted in the absence of any commercial or financial relationships that could be construed as a potential conflict of interest.

## Publisher’s note

All claims expressed in this article are solely those of the authors and do not necessarily represent those of their affiliated organizations, or those of the publisher, the editors and the reviewers. Any product that may be evaluated in this article, or claim that may be made by its manufacturer, is not guaranteed or endorsed by the publisher.

